# Successful wound closure using fibrin glue for intractable neobladder‐urethral anastomosis leakage after radical cystectomy and neobladder reconstruction

**DOI:** 10.1002/iju5.12592

**Published:** 2023-07-10

**Authors:** Keisuke Ishikawa, Masayoshi Nagata, Yuta Anno, Miki Yamagishi, Toshiyuki China, Fumitaka Shimizu, Shuji Isotani, Satoru Muto, Shigeo Horie

**Affiliations:** ^1^ Department of Urology Juntendo University Graduate School of Medicine Tokyo Japan

**Keywords:** anastomotic leakage, cystectomy, fibrin glue, neobladder reconstruction, wound closure

## Abstract

**Introduction:**

Complications of cystectomy and neobladder reconstruction such as anastomotic leakage have been reported. It is a common complication; however, most cases improve conservatively. The use of fibrin glue for fistulas has been reported, but no reports have shown its effectiveness for urinary tract anastomotic leakage. We experienced a case of intractable neobladder‐urethral anastomosis leakage after radical cystectomy and neobladder reconstruction, which was effectively managed using fibrin glue.

**Case presentation:**

A 70‐year‐old man underwent radical cystectomy and ileal neobladder reconstruction for invasive bladder cancer with urothelial carcinoma. After surgery, the urethral catheter fell off and the anastomotic leakage did not improve by adjusting the position of the urethral catheter and percutaneous nephrostomy. We closed the intractable neobladder‐urethral anastomotic leakage by injecting fibrin glue and the leakage completely disappeared.

**Conclusion:**

Injecting fibrin glue into anastomotic site can be effective in severe neobladder‐urethral anastomosis leakage.

**Abbreviations & Acronyms**
BMI: body mass index
CT: computed tomography
PCN: percutaneous nephrostomy
POD: postoperative day
RARC: robotic‐assisted radical cystectomy
TUR‐BT: transurethral resection of bladder tumor

**Keynote message**

Intractable anastomotic leakage is difficult to improve in severe neobladder‐urethral reconstruction. Conservative management may not be effective in severe cases. Treatment with fibrin glue can be effective.

## Introduction

Neobladder reconstruction was established as a standard technique for urinary diversion since the complications are less or similar to those of the ileal conduit.^1^ One of the complications of neobladder reconstruction is neobladder‐urethral anastomotic leakage, which is reported to occur in 2.1–10% of cases.^2^ A urethral catheter is recommended to be placed until the leakage disappears.^2^ Most anastomotic leakages conservatively improve; no reports have shown aggressive treatment for intractable anastomotic leakage. Herein, we report a case in which fibrin glue was effective for intractable neobladder‐urethral anastomotic leakage.

## Case presentation

A 70‐year‐old man, with no other medical history, had undergone TUR‐BT for a broad‐based papillary tumor expending spread to the bladder neck. Pathological findings of TUR‐BT revealed urothelial carcinoma, high grade, G3, pT2. The patient underwent preoperative chemotherapy with gemcitabine and cisplatin. However, it was discontinued because skin rashes appeared in the first course. It was changed to chemotherapy with methotrexate, vinblastine, doxorubicin, and cisplatin; a total of three cycles were performed.

**Fig. 1:**
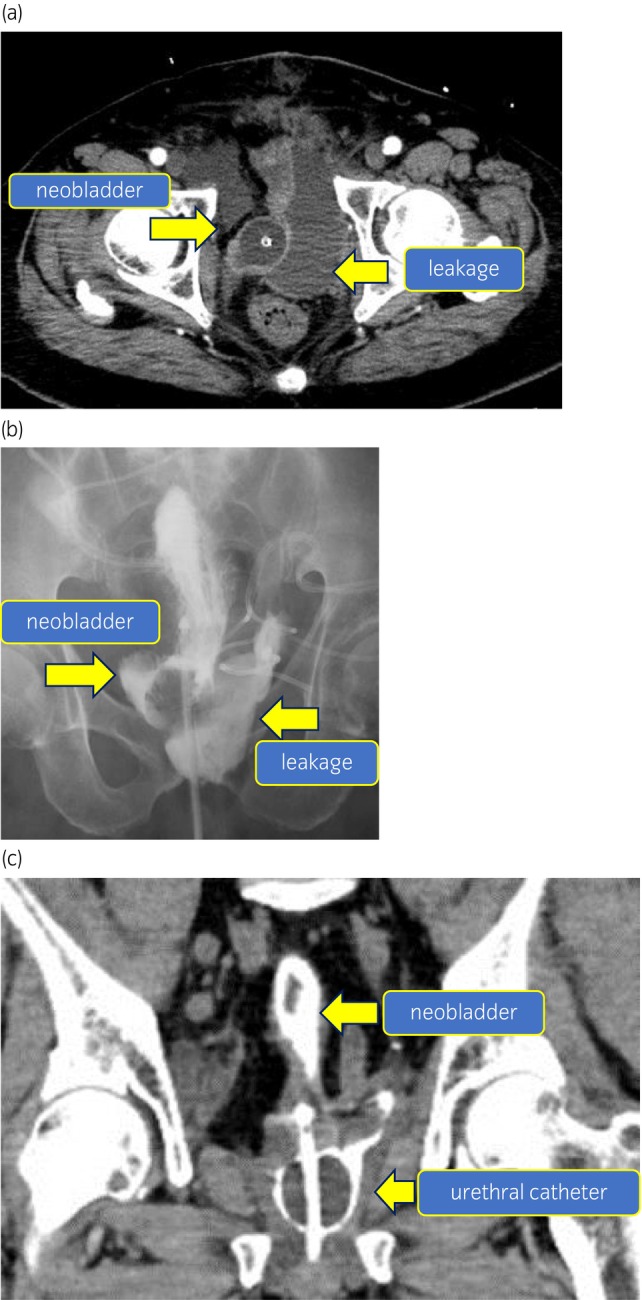
(a) CT image showing the leakage. CT scan of horizontal view for the fever examination revealed large amount of fluid collection on the left side of the urethral‐neobladder anastomosis. This may be a lymphocele infection or leakage of urine. (b) Image of leakage in cystography. Cystography reveals a large amount of leakage on the left side of the anastomosis. It reveals a neobladder‐urothelial anastomotic leakage. (c) CT image of severe leakage. CT scan of the coronary view showing urethral catheter falling off. The urethral balloon is falling off from the neobladder in a CT scan of coronary view. It suggests a severe anastomotic dehiscence of suture.

**Fig. 2:**
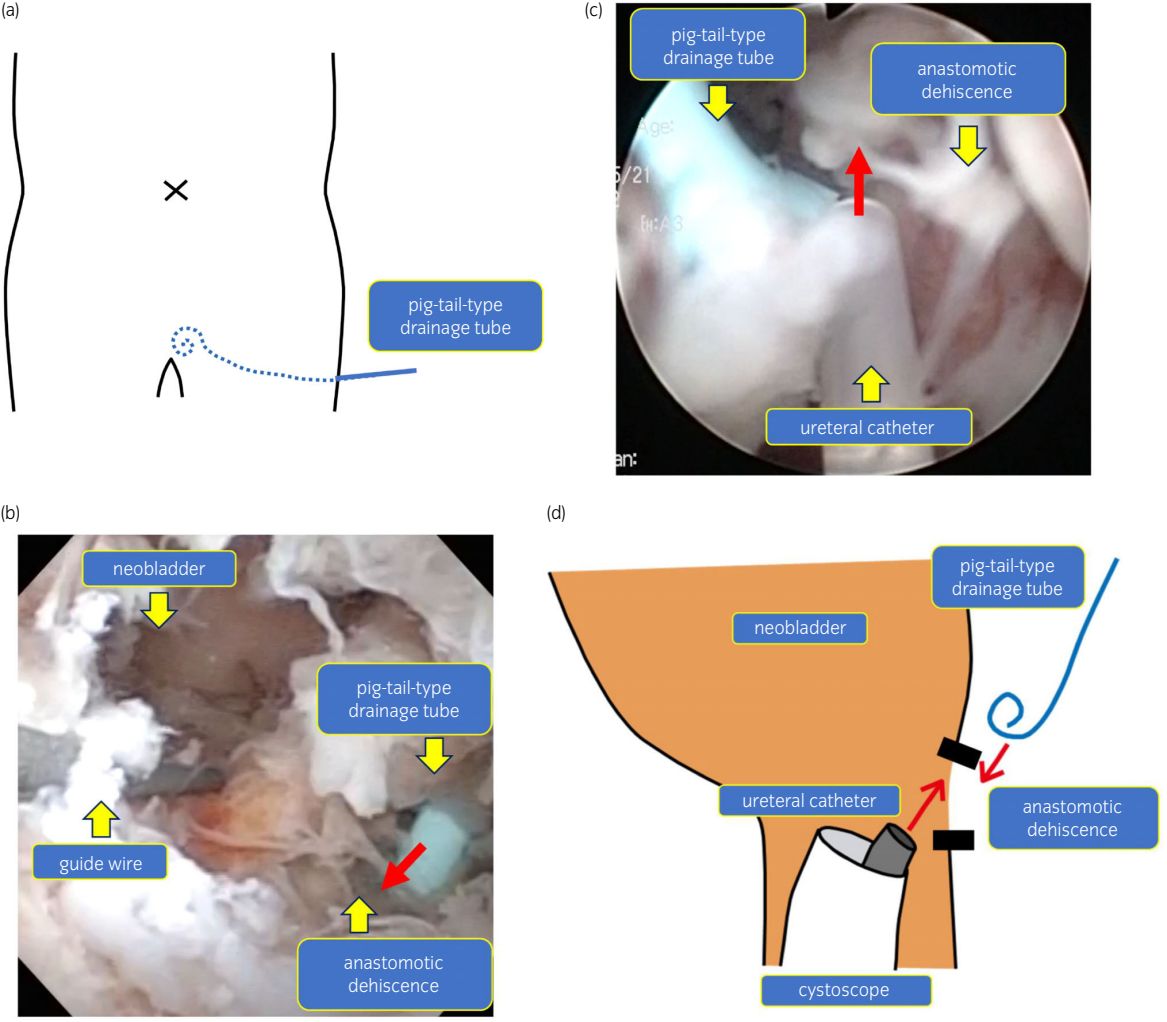
(a) Image of drainage tube position. We placed a pigtail‐type drainage tube transperineally beside the anastomosis of the neobladder. (b) Image of injecting fibrin glue with a cystoscope. We injected 10 mL of fibrin glue into the large space beside the anastomotic dehiscence from the drainage tube. (c) Image of injecting fibrin glue with a cystoscope. We sprayed 5 mL of fibrin glue around the anastomotic dehiscence from the ureteral catheter using a cystoscope. (d) Schematic diagram of injecting fibrin glue. First, we injected fibrin from the drainage tube. Second, we injected fibrin glue from the ureteral catheter using a cystoscope.

After neoadjuvant chemotherapy, we performed RARC and neobladder reconstruction by extracorporeal urinary diversion with cT2N0M0. After RARC, a small bowel segment, 55 cm in length, was resected approximately 20 cm proximal to the ileocecal valve, the ileum was arranged in J‐shape by the Studer modified technique. The afferent parachute was set to 15 cm and the rest was detubated. It was confirmed that the reservoir reached the pelvic floor after its creation, the internal urethral orifice was formed, and everting suture was performed. The ureter was anastomosed by the Wallace method. The urethral anastomosis was performed with difficulty, due to the tension created, using six singular sutures by 3‐0 absorption thread. Although a minor leakage was found during the leak test, we decided to observe. The drainage tube was placed on the pelvic floor. Immediately after surgery, we found the urethral catheter falling off spontaneously. We tried to replace it with a cystoscope. However, it was impossible because anastomosis between the ileal neobladder and urethra was separated. Thus, we reopened the abdomen and sutured it again. Only minor leakage was found during the leak test. Pathological findings revealed invasive urothelial carcinoma with glandular differentiation (pT4aN0Mx) and the surgical margin was negative. A CT performed to evaluate the fever etiology on the 11th POD showed fluid storage near the urethral anastomosis **(**Fig. 1a **)**. We suspected lymphocele infection and CT‐guided drainage was performed and a pigtail‐type drainage tube was placed **(**Fig. 2a **)**. The drainage creatinine level was 50.2 mg/dL. The cause of fever was diagnosed to be urine leakage. The leakage was also found on cystography **(**Fig. 1b **)** and the urethral catheter was lightly towed. On the 21st POD, a CT scan showed that the urethral catheter had completely fallen off the neobladder **(**Fig. 1c **)**. The position of the urethral catheter was adjusted into the neobladder under fluoroscopy immediately. Five days later, a similar incidence happened, which suggested the existence of a large space peri the neobladder‐urethral anastomosis.

**Fig. 3:**
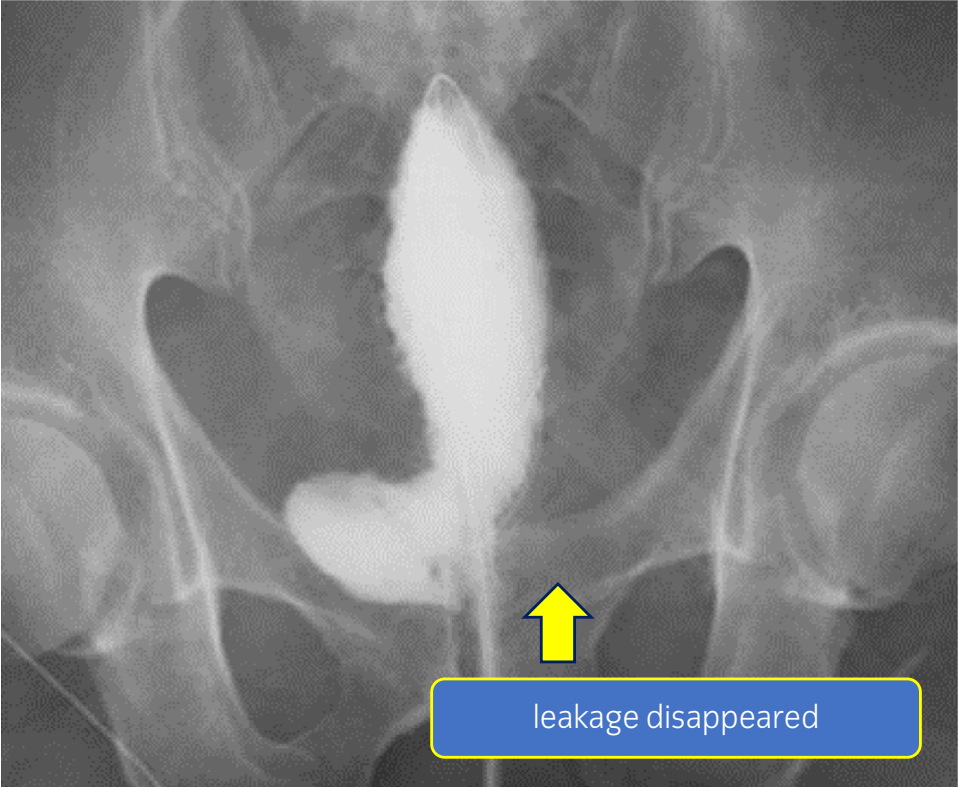
Cystography image after the disappearance of leakage. The leakage on the left side of the anastomosis has disappeared.

On the 33rd POD, we performed bilateral PCN and confirmed the anastomotic dehiscence of suture in the 1 to 6 o'clock direction with cystoscopy. Leakage did not improve at all on the 40th POD, and we decided to use fibrin glue to treat the severe anastomotic leakage. Since the patient's nutritional status had already improved postoperatively and it would take a long time to close the anastomotic dehiscence, thereby we decided to actively intervene. On the 42nd POD, we observed the anastomotic dehiscence and the pigtail‐type drainage tube using a cystoscope. Then, we injected 10 mL of fibrin glue into the large space beside the anastomosis from the drainage tube **(**Fig. 2b,d**)**. Finally, we sprayed 5 mL around the anastomotic dehiscence of suture from the ureteral catheter using a cystoscope **(**Fig. 2c,d**)**. The drainage tube was immediately removed to avoid clogging with the fibrin glue. On the 54th POD, we cystographically confirmed no leakage **(**Fig. 3 **)**; we then removed the bilateral nephrostomy catheter. The patient was discharged after 61 days of hospitalization. Urinary incontinence continued for about 2 months after discharge from the hospital, though improved thereafter and the patient was managed with spontaneous voiding by abdominal pressure. It has been 1 year since then without any leakage, anastomosis stricture, or recurrence of cancer.

## Discussion

Urinary leakage is a relatively common complication after ileal neobladder reconstruction. To prevent the leakage, it is recommended to conservatively follow‐up, use meticulous closure of conduit/pouch with seromuscular sutures, ensure good spatulation, and maintain stent or drainage tube placement. In most cases, it can be treated conservatively.^2^ There have been no reports of treatment with adhesives for intractable neobladder‐urethral anastomotic leakage. Many reports describe the use of sealing agents as a method of closing fistulas, although there were no anastomotic leakages. Fibrin glue is a tissue adhesive that induces a coagulation reaction by mixing fibrinogen and thrombin, and it is highly safe because it utilizes a normal biochemical reaction. In addition to local hemostasis, it is used to seal wounds and fistulas, and for nephroplasty, fixation of ureteral anastomosis, urethral skin, and bladder colon fistulas.^3^, ^4^ It has also been reported that transurethral injection of fibrin glue improved urinary leakage after partial nephrectomy.^5^ The injected fibrin glue remains in a solid‐state for approximately 24 h and then denatures into a gel state in approximately 5 days. No reports have been shown so far that fibrin glue remaining in the urethra causes clinical problems such as urinary stones.

In this case, to prevent anastomotic dehiscence of suture, we should have considered that anastomotic tension should be avoided by making relaxing incision on mesentery during surgery. Initially, when we diagnosed that the degree of anastomotic leakage was so severe, although placing percutaneous pouch‐stormy was considered as a drainage method to confer secure drainage of neobladder, bilateral PCN was chosen due to invasiveness. However, no improvement was observed with temporary changes in the urinary tract, thus we judged that it was difficult to improve the leakage conservatively. Finally, the anastomotic leakage could be repaired and healed by injecting fibrin glue. No complications have occurred, including stenosis and foreign body formation until now.

## Conclusion

Treatment with fibrin glue may be effective if it is challenging to improve intractable neobladder‐urethral anastomotic leakage by conservative treatment.

## Author contributions

Keisuke Ishikawa: Conceptualization; data curation; investigation; writing – original draft. Masayoshi Nagata: Data curation; methodology; supervision; writing – review and editing. Yuta Anno: Data curation. Miki Yamagishi: Data curation. Toshiyuki China: Data curation; methodology; project administration. Fumitaka Shimizu: Data curation; methodology. Shuji Isotani: Data curation. Satoru Muto: Data curation. Shigeo Horie: Conceptualization; data curation; investigation; methodology; project administration; supervision; validation; visualization; writing – review and editing.

## Conflict of interest

The authors declare no conflict of interest.

## Approval of the research protocol by an Institutional Reviewer Board

Not applicable.

## Informed consent

Not applicable.

## Registry and the Registration No. of the study/trial

Not applicable.
